# Probing the Structural Dynamics of the Catalytic Domain of Human Soluble Guanylate Cyclase

**DOI:** 10.1038/s41598-020-66310-4

**Published:** 2020-06-11

**Authors:** Rana Rehan Khalid, Arooma Maryam, Osman Ugur Sezerman, Efstratios Mylonas, Abdul Rauf Siddiqi, Michael Kokkinidis

**Affiliations:** 1 Department of Biosciences, COMSATS University, Islamabad, 45550 Pakistan; 2 Department of Biology, University of Crete, 70013 Heraklion, Greece; 3 Department of Biostatistics and Medical Informatics, Acibadem M. A. A. University, Istanbul, 34752 Turkey; 4 Department of Pharmaceutical Chemistry, Biruni Universitesi, Istanbul, 34010 Turkey; 5 Institute of Molecular Biology and Biotechnology, Foundation for Research and Technology-Hellas (IMBB-FORTH), 70013 Heraklion, Greece

**Keywords:** Computational models, Molecular conformation, Bioinformatics, Molecular modelling, GTP-binding protein regulators

## Abstract

In the nitric oxide (NO) signaling pathway, human soluble guanylate cyclase (*h*sGC) synthesizes cyclic guanosine monophosphate (cGMP); responsible for the regulation of cGMP-specific protein kinases (PKGs) and phosphodiesterases (PDEs). The crystal structure of the inactive *h*sGC cyclase dimer is known, but there is still a lack of information regarding the substrate-specific internal motions that are essential for the catalytic mechanism of the *hs*GC. In the current study, the *hs*GC cyclase heterodimer complexed with guanosine triphosphate (GTP) and cGMP was subjected to molecular dynamics simulations, to investigate the conformational dynamics that have functional implications on the catalytic activity of *hs*GC. Results revealed that in the GTP-bound complex of the *h*sGC heterodimer, helix 1 of subunit α (α:h1) moves slightly inwards and comes close to helix 4 of subunit β (β:h4). This conformational change brings loop 2 of subunit β (β:L2) closer to helix 2 of subunit α (α:h2). Likewise, loop 2 of subunit α (α:L2) comes closer to helix 2 of subunit β (β:h2). These structural events stabilize and lock GTP within the closed pocket for cyclization. In the cGMP-bound complex, α:L2 detaches from β:h2 and establishes interactions with β:L2, which results in the loss of global structure compactness. Furthermore, with the release of pyrophosphate, the interaction between α:h1 and β:L2 weakens, abolishing the tight packing of the binding pocket. This study discusses the conformational changes induced by the binding of GTP and cGMP to the *h*sGC catalytic domain, valuable in designing new therapeutic strategies for the treatment of cardiovascular diseases.

## Introduction

Guanylate cyclase (GC) exists as membrane-bound particulate guanylate cyclase (pGC) and cytoplasmic soluble guanylate cyclase (sGC) in mammalian tissues. Both these forms of the sGC utilize guanosine triphosphate (GTP) as a substrate and metal ions (Mg^2+^, Mn^2+^) as cofactors to stimulate the release of inorganic pyrophosphate and promote cyclization at the α-phosphorus atom to form 3′,5′-cyclic guanosine monophosphate (cGMP)^1^. Nitric oxide (NO) and hormones modulate the activity of sGC and pGC respectively. It is now established that cGMP binds to various downstream signal transduction effector proteins and ligand-gated ion channels to regulate a multitude of physiological processes and pathological conditions such as cerebellar motor control, cardiac failure, pulmonary hypertension, erectile dysfunction, gut peristalsis and neurodegeneration^2–5^.

Soluble guanylate cyclase (sGC) is a heterodimeric protein consisting of α and β subunits. In human, four different subunits (α_1_, α_2_, β_1_ and β_2_) exist which assemble to make α_1_β_1_, α_2_β_1_ and α_1_β_2_ heterodimer isozymes while α_1_α_1_ & β_1_β_1_ homodimers also exist. Out of all these isoforms, only two sGC isozyme, α_1_β_1_ and α_2_β_1_, are well characterized in human^1,6^. α_1_β_1_ is an abundantly expressed cytosolic protein and a well-established member in the NO signaling cascade of mammals and insects. In contrast, the α_2_β_1_ isoform is membrane-associated and despite common ligand-binding features they respond differently and play distinct roles in the body. Although sGC is most ubiquitously expressed in lung, brain, muscle, spleen, brain and heart its distribution across tissues is isoform-specific. Levels of cGMP at a particular site in the human body can be regulated by modulating the expression of specific guanylyl cyclase isoforms^7^.

Each subunit forms a multi-domain protein consisting of four globular domains. The N-terminus of the β_1_ subunit harbors a heme-NO binding (H-NOX) domain; a conserved structural region critical for NO and O_2_ binding. The corresponding region on the α_1_ subunit is known as pseudo-HNOX domain because it lacks the heme-binding motif^8–10^. Binding of NO to the heme group of the HNOX domain induces a conformational switch in sGC. The NO-binding signal is disseminated to the catalytic region through the Per/Arnt/Sim (PAS) and long helical coiled coil domains that form the dimeric backbone of the sGC quaternary structure. Both domains are known to be involved in NO-induced signal transduction and heterodimerization^8,11,12^. The C-terminal catalytic domains of the α_1_ and β_1_ subunits are responsible for the cyclization and synthesis of cGMP from GTP^13,14^.

The cyclase domain of sGC is a closely related homolog of the catalytic domains of soluble adenylate cyclase (sAC) and particulate guanylate cyclase (pGC). Soluble guanylate cyclase (sGC) is categorized into the class III purine nucleotidyl cyclase family like all aforementioned closely related homologs^15,16^. All these proteins convert purine-based ribonucleotide triphosphates (GTP, ATP) to their respective cyclic ribonucleotide monophosphates (cGMP, cAMP) in a similar manner^17^.

The three dimensional (3D) structures of the catalytic domains of sGC in apo-form (Protein Data Bank (PDB) ID: 3UVJ)^14^ and the ATP-bound complex of sAC in *Rattus norvegicus* (PDB ID: 1CJK)^18^ reveal a highly conserved secondary structure organization of the binding pockets. The catalytic domain of both proteins adopts a wreath-like head to tail orientation_._ It lies at the dimeric interface of the α_1_ and β_1_ subunits and it is composed of residues from both catalytic subdomains^19^. The substrate binding site of the cyclase domain consists of the conserved D486 and D530 residues from the α_1_ subunit and 548 N, 551 S, 552 R residues from the β_1_ subunit that establish electrostatic interactions with the phosphate moiety of GTP and metals^14^.

In human, both homodimeric and heterodimeric isoforms of the sGC cyclase domain in the apo-form have been resolved crystallographically but not the GTP-bound heterodimeric form^1,14^. The catalytic mechanism of the sGC cyclase domain is inferred from the crystal structures of the active, ATP-bound sAC complexes from human and *Rattus norvegicus* but no direct information of the active *h*sGC cyclase domain in complex with GTP is available.

To investigate the comparative dynamics behavior of the apo-, GTP- and cGMP-bound *h*sGC cyclase-α1β1 heterodimers, we carried out *in silico* structural analysis and molecular dynamics (MD) simulations. Expansive structural information concerning conserved active site motifs and ribonucleotide triphosphate substrate binding residues was gleaned from the sAC catalytic domain by superposing it with the heterodimeric *h*sGC catalytic domain complexes. The binding conformation of ATP from sAC was used to model the GTP- and cGMP-bound *h*sGC catalytic domain complexes. The complexes were then subjected to a comprehensive MD simulation analysis in order to monitor the stability and binding modes pattern of catalytic interface residues. Furthermore, principal component analysis was performed to identify structural transitions in the presence of GTP or cGMP that contribute significantly to the opening and closing of the wreath-like *h*sGC cyclase heterodimer complex. Finally, we discuss the pre- (GTP-bound *h*sGC cyclase domain) to post-catalysis (cGMP-bound *h*sGC cyclase domain) structural transition.

## Results

**Figure 1:**
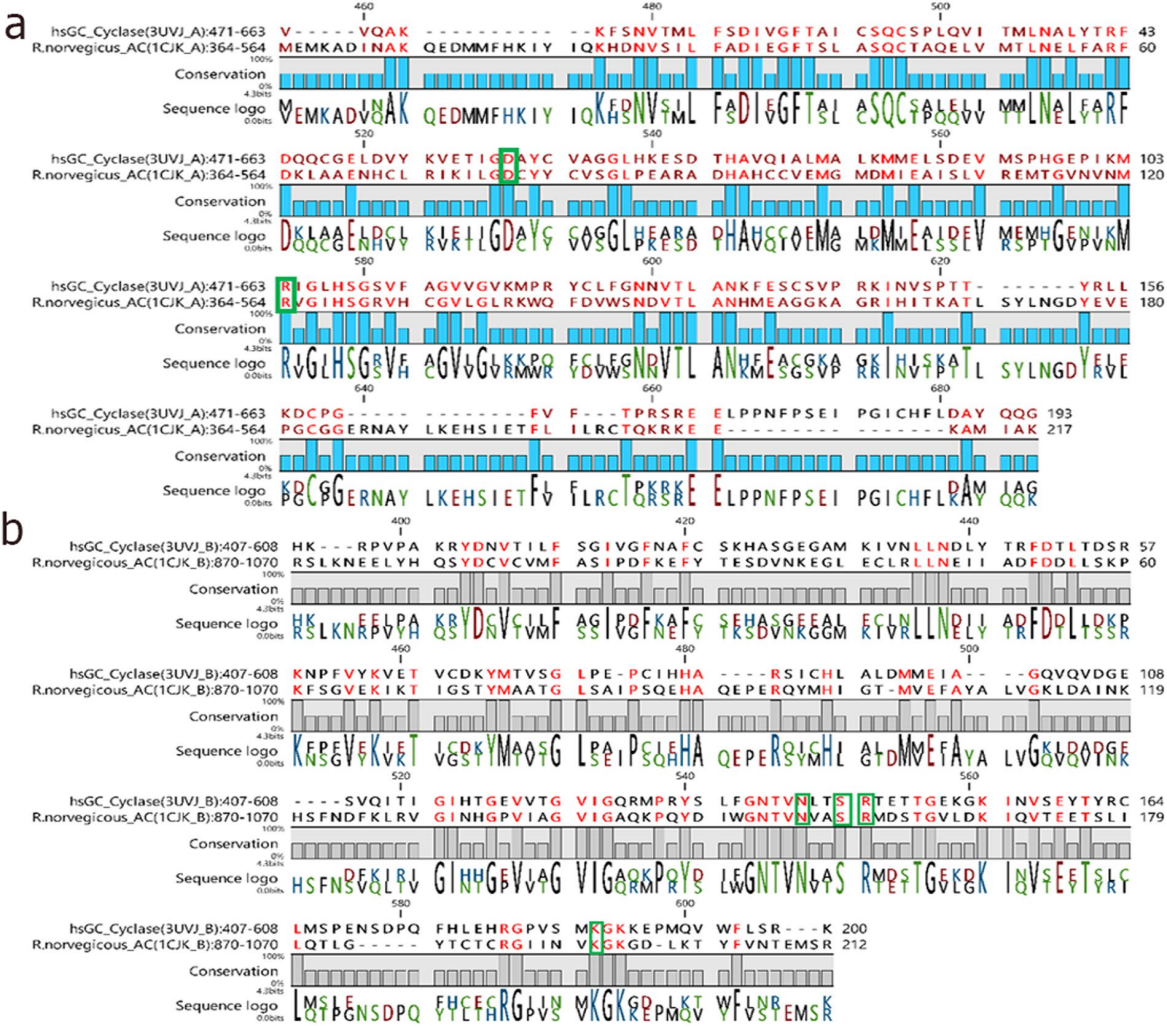
Sequence alignment of the α (**a**) and β (**b**) chains of the human soluble guanylate cyclase heterodimer (*h*sGC) with the closely related *R.norvegicus* adenylate cyclase homodimer (AC). Conserved catalytically active residues are highlighted with a green box.

Pairwise sequence alignment (Fig. 1) of the *R. norvegicus* AC with the *h*sGC cyclase domain revealed that their heterodimeric chains share >30% sequence identity and >50% sequence similarity.

### Molecular docking

**Figure 2:**
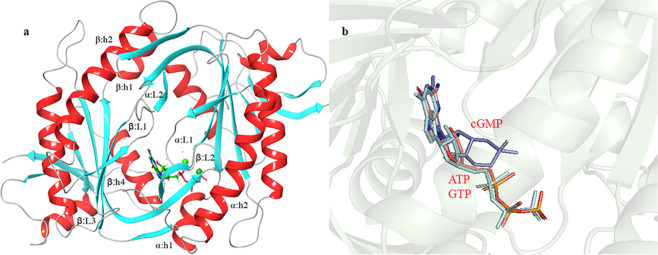
(**a**) The *h*sGC catalytic dimer. Conserved secondary structure elements of the *h*sGC catalytic dimer are shown in red and cyan. (**b**) docked GTP (pink) and cGMP (blue) illustrate the very similar orientation to the ATP (cyan) in *R.norvegicus* AC.

The crystal structure of the human heterodimeric *h*sGC cyclase domain in the apo-form is available at the Protein Data Bank (PDB ID: 3UVJ). The Glide XP module was employed to dock the GTP and cGMP molecules within the binding pocket of the *h*sGC cyclase domain. The docking protocol was validated by docking ATP within the *R.norvegicus* sAC catalytic domain and superposing with the already reported ATP-bound complex of sAC (PDB ID: 1CJK)^18^ (Supplementary Fig. 1a). Binding pocket residues (αD396, αD441, βN1025, βR1029 and βK1065), involved in the binding of nucleotide tri-phosphate (NTP) within the active site of adenylate cyclase, are conserved at positions αD486, αD530, βN548, βR552 and βK593 of *h*sGC (Supplementary Fig. 1b). Conserved catalytic residues highlighted in Fig. 1. The conformation of the Glide-docked ATP was very similar to the experimental one with an RMSD 0.4 Å. The ATP-binding pocket grid coordinates that were extracted from the *R.norvegicus* sAC catalytic domain were used for the docking of GTP and cGMP to the sGC cyclase domain. Secondary structure elements such as helix1 (h1), helix2 (h2), β hairpin loop1 (β3L1β4), β hairpin loop2 (β6L2β7) of α and β chains constitute the ligand-binding pocket and the dimeric interface of the *h*sGC catalytic dimer (Fig. 2a).

The Glide XP dock scores for GTP and cGMP are −6.718 and −7.295 kcal/mol respectively.

**Figure 3:**
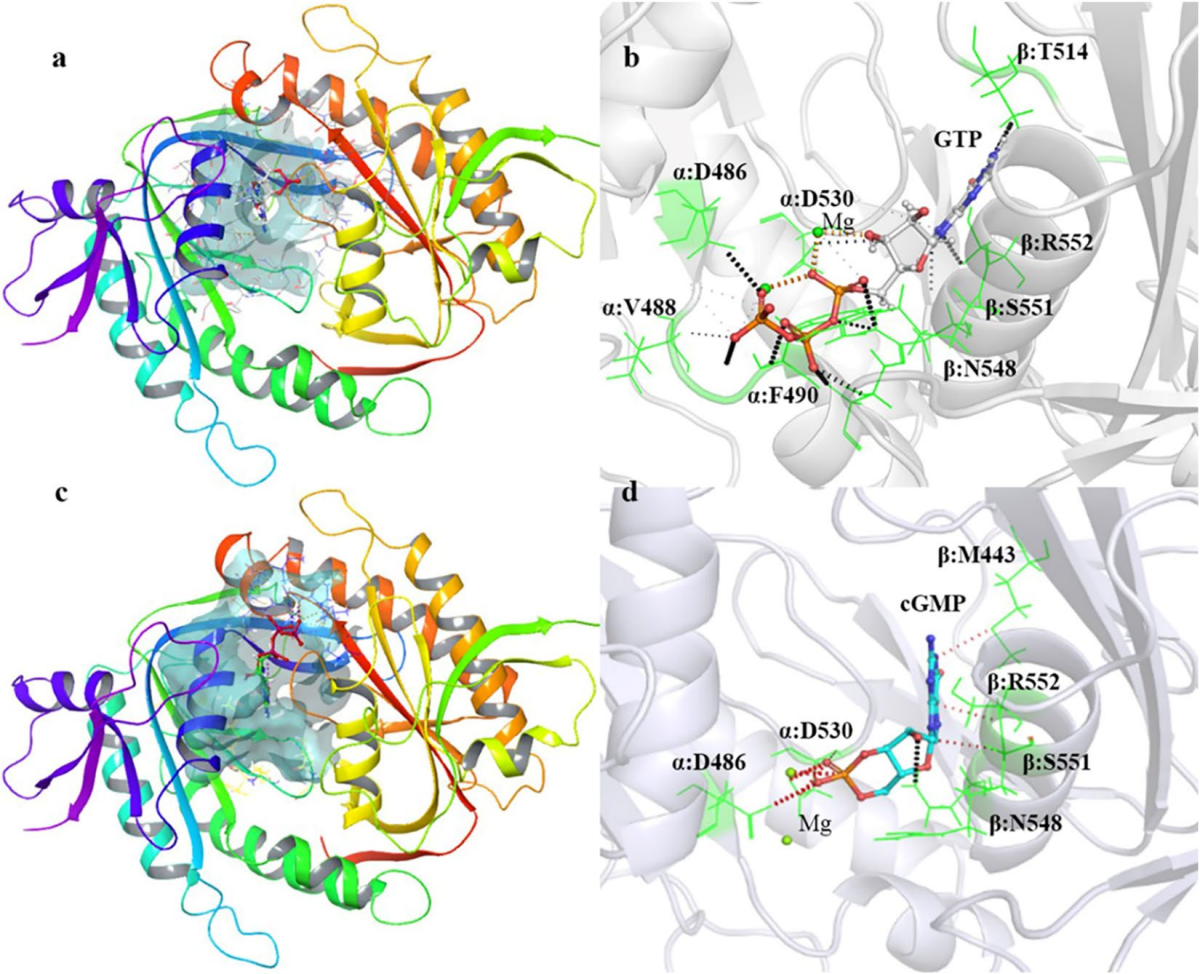
(**a**) Docked complex of GTP with the *h*sGC catalytic heterodimer. The active site of the catalytic heterodimer is shown in transparent cyan. (**b**) Interactions of GTP (shown as ball and stick) with the active site residues (shown as green colored lines). Hydrogen bonds are shown as black dotted lines (dense: strong; light: weak) and van der Waals interactions as orange dotted lines. (**c**) Docked complex of cGMP with the *h*sGC catalytic heterodimer. The active site of the catalytic heterodimer is shown in transparent cyan. (**d**) Interactions of cGMP (shown as ball and stick) with active site residues (shown as green colored lines). Hydrogen bonds are shown as black dotted lines (dense: strong; light: weak) and van der Waals interactions as red dotted lines.

The GTP- and cGMP-docked complexes were again superposed to the ATP-bound *R. norvegicus* sAC catalytic domain to validate the best docked conformations (Fig. 2b). In the GTP-*h*sGC cyclase domain complex highlighted in Fig. 3a. Residues D486, V488, F490 and D530 from α chain and N548, S551, R552 and T514 from β chain are encapsulating the GTP molecule within the catalytic pocket through various molecular interactions (Fig. 3b). Of all the GTP-interacting residues, D486 and D530 from the α chain of the *h*sGC cyclase showed Mg^2+^-mediated interactions with the GTP. Beside these conserved residues, F490 showed hydrogen bond interactions and V488 weak van der Waals interactions with the GTP molecule. All β-chain residues i.e. N548, S551, R552 and T514 formed a dense network of hydrogen bonds with the GTP molecule (Fig. 3b).

Similarly, cGMP adopted a similar orientation within the dimeric pocket of the *h*sGC cyclase domain (Fig. 3c) but formed interactions with only six binding pocket residues. The Mg^2+^-mediated ionic interactions of cGMP with D486 and D530 of the α chain persisted while only a single hydrogen bond with N548 and three van der Waals interactions with S551, R552 and M443 of the β chain were observed upon binding of cGMP (Fig. 3d).

Similar indirect interactions of the *h*sGC cyclase α chain residues (D486 and D530) with GTP and cGMP through two Mg^2+^ ions have also been reported in the adenylate cyclase-ATP complex^12,14^. This suggests a conserved role of these residues in the activity of the class III purine nucleotidyl cyclase family of enzymes. In addition to this, N548, S551, and R552 from the *h*sGC cyclase α chain are also conserved and involved in the stabilization of the GTP and cGMP molecules in the cyclase domain.

### Stability analysis of MD simulations

#### Root-mean-square deviation

Stability analysis was carried out for the apo, GTP- and cGMP-bound cyclase αβ complexes to identify the changes in trajectories. The root-mean-square deviation (RMSD) and root mean square fluctuation (RMSF) were calculated for the backbone atoms of all systems using the initial apo crystal structure as a reference. Afterwards, the radius of gyration (Rog) was also calculated for all atoms of our systems.

**Figure 4:**
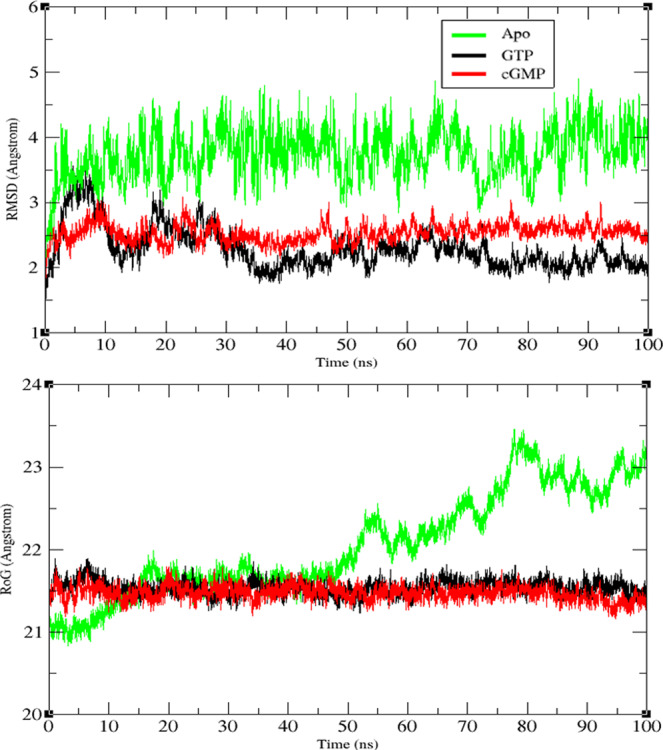
Root mean square deviation (RMSD) and radius of gyration (Rog) of the *h*sGC catalytic dimer in the apo form and in complex with GTP and cGMP over the course of 100 ns simulations.

The RMSD analysis showed that the system with the highest structural variation was the apo system with an average RMSD of 3.9 Å and a high fluctuation rate during the simulation (Fig. 4).

The GTP-bound sGC cyclase heterodimer underwent a large structural change (RMSD 3.5 Å) at the beginning of the simulation (5–7 ns) and gradually attained stability at 20–100 ns with an average RMSD of 2.1 Å. The difference of RMSD observed during the first 20 ns is possibly associated with the closed pocket conformation of the cyclase heterodimer. In next 30–100 ns, the closed pocket conformation becomes stable which helps in the gradual decrease in RMSD to 2.1 Å.

Interestingly, the cGMP-bound cyclase-αβ heterodimer showed an average RMSD of 2.5 Å that remained relatively stable throughout the entire simulation. Of note is the 0.6 Å RMSD difference observed between the GTP- and cGMP-bound systems during the last 20 ns of the simulations that may be attributed to the opening and closing of the wreath-like cyclase heterodimer. The presence of GTP at the substrate binding site stabilizes the dimeric pocket of the *h*sGC cyclase heterodimer with a robust network of interactions that are partially absent in the presence of cGMP where a slight opening of the pocket is observed.

### Radius of gyration (Rog)

The radius of gyration is another stability indicator which represents the mass-weighted RMS distance of a group of atoms from their common center of mass and is used to estimate the global dimension of proteins.

The analysis showed that both substrate-bound complexes were stable during the simulation. The Rog values ranged between 21–21.5 Å, signifying moderate fold movement among GTP/cGMP bound systems (Fig. 4). Results suggest that the GTP- and cGMP-bound dimeric cyclase remains compact over the course of simulation while the apo-cyclase system experiences broader configurational variations up to 23.3 Å. The Rog analysis results are consistent with the RMSD analysis.

### Root mean square fluctuation

**Figure 5:**
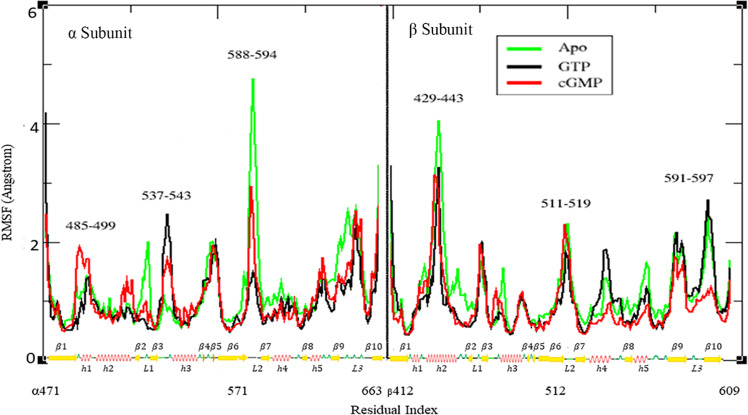
Root mean square fluctuation (RMSF) of the *h*sGC catalytic dimer in the apo form and in complex with GTP and cGMP. The x–axis of the graph is representing the sequence of the chains α and β with the secondary structure elements highlighted accordingly while the y-axis shows the residual fluctuation in Angstroms (Å).

The root-mean-square fluctuation (RMSF) analysis is indispensable for the characterization of structural fluctuations within local regions of the *h*sGC cyclase dimer after ligand (GTP, cGMP) binding. The substrate-anchoring regions of the *h*sGC catalytic dimer that interact with GTP and cGMP include α:h1, β hairpin loop α:L1, β hairpin loop β:L2 and β:h4. During simulations we monitored conformational changes in these important secondary structure elements. RMSF analysis of the *h*sGC cyclase dimer in the absence of ligand revealed 1.4 Å, 4.8 Å and 2.3 Å deviations in α:h1, α:L2 and β:L2 regions, respectively, indicating high conformational flexibility (Fig. 5).

In contrast to the apo state, the GTP- and cGMP-ligated complexes showed fewer residual transitions. The GTP-and the cGMP-bound *h*sGC cyclase αβ systems showed distinct patterns of RMSF peaks different from each other. In the human sGC catalytic dimer complexed with GTP, structurally significant regions, especially α:h1, α:L2 and β:L2 showed RMSF peaks of 1.4 Å, 1.5 Å and 1.8 Å, respectively, indicating a robust increase of the stability of the system upon GTP binding compared to the apo-state (Fig. 5). In the presence of cGMP, the α:h1, α:L2 and the β:L2 regions showed RMSF peaks at 2 Å, 2.5 Å and 2.3 Å, respectively, indicating a less compact arrangement compared to the GTP-bound system (Fig. 5).

The difference of the RMSF peaks in structurally critical regions of the human cyclase dimer in the presence of GTP indicates a more stable and/ or rigid organization of the secondary structure elements potentially favoring GTP binding and catalysis. The enhanced flexibility of the cGMP-bound system might be associated with the release of cGMP, after GTP catalysis.

### Hydrogen bond analysis of the ligand-bound complexes

Both cGMP and GTP bound within the cyclase pocket of the *h*sGC catalytic domain were subjected to hydrogen bond analysis to assess the occurrence of hydrogen bonds between the ligand and the side chain atoms of the binding pocket residues over the 50 ns simulations. The occupancy of hydrogen bonds throughout the simulation signifies the contribution of hydrogen bond interactions to the structural stability of the complexes.

The GTP-bound *h*sGC catalytic dimer showed a greater number of hydrogen bond interactions compared to the cGMP system (Table 1). The GTP- and cGMP-mediated hydrogen bond network can be mediated through the heterocyclic guanine (N1, N2, N3, O6, N7), ribose sugar (O3′, O2′, O5, O3), and phosphate (O1B, O2A, O2B, O1G, O2G, O3G, O7) group atoms (some donor-acceptor pairs in Table 1 have low occupancy but they are included either because the cumulative effect on the same residue is significant or they are residues important in the subsequent analyses).

**Table 1:** Comparison of the GTP- and cGMP-bound catalytic heterodimers hydrogen bond interactions of the nucleotidyl substrates with the binding pocket residues.

Ligands	Acceptor	Donor	Percentage Occupancy	Average Distance	Average Angle
	GTP@O1B GTP@O3G β:E473@O GTP@O2B GTP@O1G β:E473@O GTP@O2G GTP@O2A GTP@O6 GTP@O3′ GTP@O3G GTP@O2′ GTP@N7 GTP@O2A GTP@O3G GTP@O2G β:T474@O	α:T491@O α:G489@N GTP@N2 α:F490@N α:R574@N GTP@N1 β:K593@NZ β:N548@N β:L542@N β:S551@O α:R574@N β:S551@O β:L542@N β:R552@N β:K593@N α:R574@N GTP@N2	99.64 73.90 66.78 59.42 52.20 44.69 40.70 30.73 26.79 17.69 14.87 10.84 9.15 6.90 6.65 6.62 5.75	2.62 2.82 2.84 2.86 2.83 2.87 2.78 2.81 2.88 2.76 2.86 2.77 2.92 2.78 2.77 2.85 2.88	167.51 160.59 155.18 150.52 157.66 150.11 156.20 159.06 162.38 161.33 160.31 158.10 157.74 156.71 156.42 155.48 153.39
	GMP@O1 GMP@O5 β:V475@O GMP@O7 β:V475@O GMP@O3 GMP@N3	β:V475@N β:S551@O GMP@N1 α:D530@N GMP@N2 β:S551@O β:S551@O	65.29 32.67 21.23 17.37 0.20 0.16 0.11	2.86 2.80 2.80 2.90 2.91 2.75 2.86	159.68 162.38 162.09 160.61 149.73 159.09 162.96

**Figure 6:**
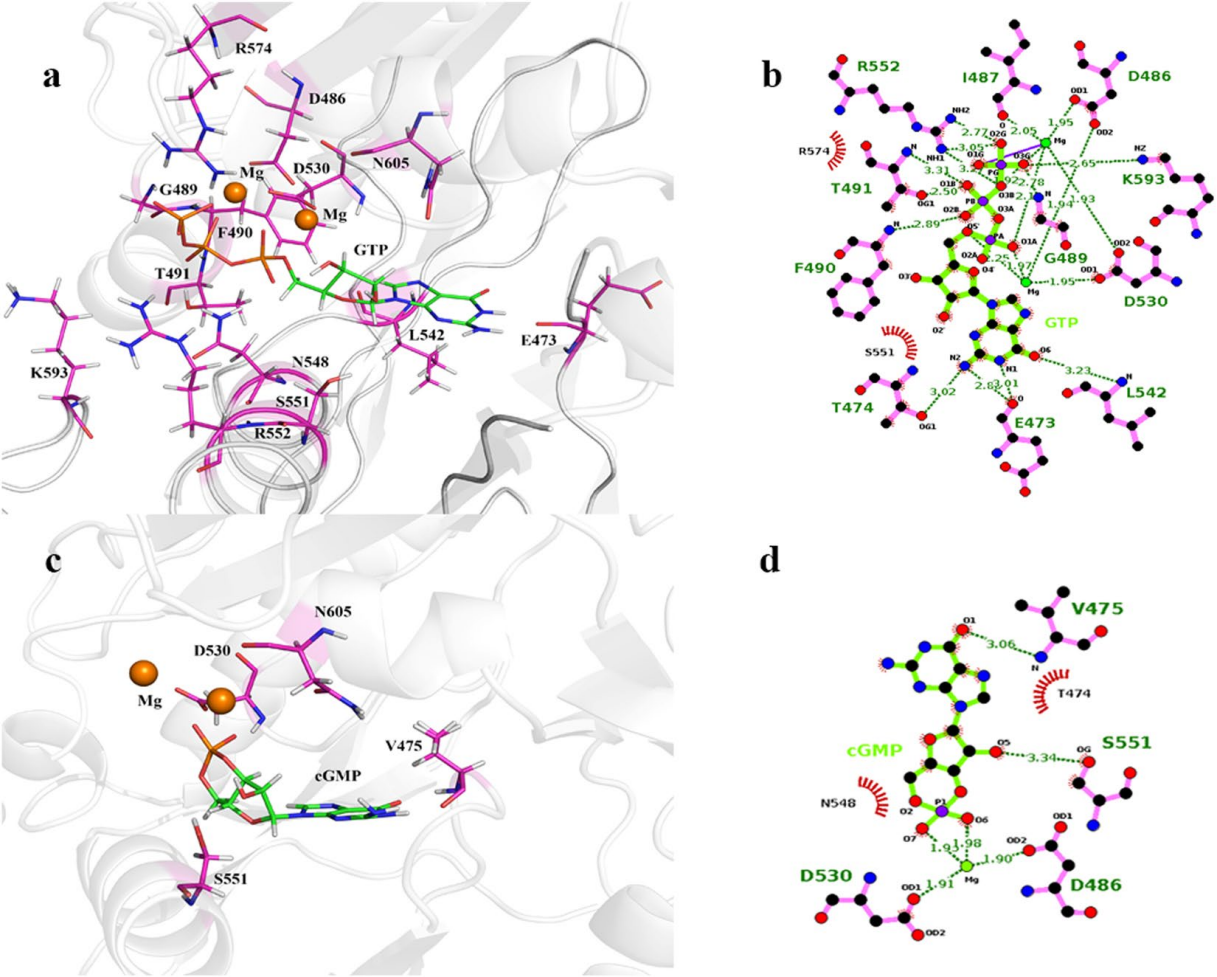
(**a**) Graphical representation of the binding pocket residues (purple) of *h*sGC that interact with GTP throughout the 50 ns MD simulation. (**b**) LigPlot two-dimensional (2D) ligand interaction diagram of the hydrogen bond interactions between the catalytic pocket residues of the *h*sGC heterodimer and GTP. (**c**) Graphical representation of the binding pocket residues (purple) of *h*sGC that interact with cGMP throughout the simulation. (**d**) LigPlot two-dimensional (2D) ligand interaction diagram of the hydrogen bond interactions between the catalytic pocket residues of the *h*sGC heterodimer and cGMP.

Among the aforementioned groups, the nitrogenous base atoms of GTP interact with E473, L542, while the nitrogenous base of cGMP interacts only with V475. The 5′ carbon sugar group atoms established a robust interaction with S551 consistent throughout the 50 ns simulation in both GTP- and cGMP-bound complexes. The phosphate group atoms of GTP fostered a complex hydrogen bond network with T491, G489, F490, R574, K593, N548 and R552 (high to low fractional value), partially absent in case of cGMP because of the release of pyrophosphate during the catalysis of GTP to cGMP. The binding pocket residues of the *h*sGC catalytic dimer that are lying in close proximity to GTP and interact with it through non-covalent interactions are shown in Fig. 6a,b. The electrostatic interactions of the GTP-bound *h*sGC cyclase dimer are consistent with previous findings^7,14^.

In a study reported by Kleinboelting *et al*., 2014, aspartate residues critical for metal ion binding and indirect interactions of NTPs with the NSR motif (Asn, Ser and Arg) are also conserved in the current study^19^. In addition to the conserved residues, 489 G, 490 F, 491 T at helix 1(h_1_), R574 at β-sheet 5 (β_5_) of the α chain and 473E at β-hairpin loop 1(β3L1β4), 542 L at β-sheet 7 (β7) and 593 K at β-hairpin loop 3 (β9L3β10) of the β chain are forming a hub that plays a key role in the encapsulation of GTP within the cyclase αβ dimer for the cyclization event.

The conformation of the binding pocket is determined by four critical structural regions; α:h1, α:β5, β:h4, β:L3. These secondary structure elements are forming the dimeric interface of the sGC catalytic heterodimer. The highest hydrogen bond occurrence in the GTP-bound cyclase αβ complex might assist these structural components to attain a close pocket conformation. Conversely, in the cGMP-ligated cyclase αβ system the reduced number of hydrogen bond interactions at the dimeric interface results in the opening of the cyclase pocket (Fig. 6c,d).

### Substrate-mediated protein-protein interactions in the *hs*GC αβ cyclase dimer

**Figure 7:**
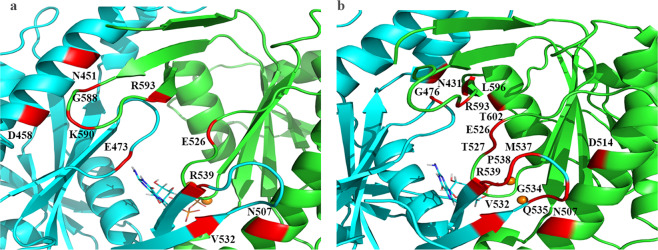
(**a**) Graphical representation of the GTP-bound *h*sGC catalytic dimer showing a closed-state conformation. Interacting residues of α (green) and β (cyan) chain are highlighted in red. (**b**) Graphical representation of the cGMP-bound *h*sGC catalytic dimer showing an open-state conformation. Interacting residues of α (green) and β (cyan) chain are highlighted in red.

To understand how local changes upon GTP and cGMP binding to the cyclase pocket induce large scale conformational changes in the structure, hydrogen bond analysis of the dimeric interface residues was carried out. In the GTP-bound system, N507 at helix 2 (h2), E526 at loop 1 (L1), G588, K590 at loop 2 (L2), R593 at β-sheet 7 (β7) of the α chain are interacting through hydrogen bonds with V532 at β-sheet 6 (β_6_), R539 at β-sheet 7 (β7), E473 at loop 1 (L1), N541 and D548 at helix 2 (h2) of the β chain (Fig. 7). Occupancies, average distances and average angles of the hydrogen bonds between the dimeric interface residues are shown in Table 2. In the GTP-bound *h*sGC cyclase, the abundance of interactions in the dimeric interface may contribute to the tight packing of the cyclase binding pocket and the catalysis of GTP.

**Table 2:** Comparison of the hydrogen bond interdomain protein-protein interactions of GTP- and cGMP-bound cyclase complexes.

Complexes	Acceptor	Donor	Percent Occupancy	Average Distance	Average Angle
Cyclase-GTP	α:E526@O β:E473@O β:D458@O β:N451@O β:V532@O	β:R539@N α:R593@N α:K590@N α:G588@N α:N507@N	87.22 74.77 53.40 43.01 27.74	2.80 2.82 2.90 2.90 2.88	158.35 158.39 165.89 154.60 161.13
Cyclase-cGMP	α:D514@O β:M537@O β:P538@O α:N507@O α:R593@O α:T527@O β:N431@O β:V532@O α:E526@O	β:Q535@N α:T527@O α:R593@N β:G534@N β:G476@N β:R539@N α:T602@O α:N507@N β:R539@N	81.62 80.29 56.53 51.60 35.17 30.07 25.37 25.37 23.88	2.83 2.74 2.81 2.86 2.88 2.83 2.75 2.87 2.79	159.18 162.54 152.30 153.87 153.52 150.91 162.06 164.44 155.82

The number of inter-residue interactions between the α and β chains of the cGMP-bound *h*sGC was higher, with only N507 at helix 2 (h2), E526 at loop 1 (L1), and R593 at β-sheet 7 (β7) of the α chain interacting with V532 at β-sheet 6 (β6), R539 at β-sheet 7 (β7) and G476 at loop 1 (L1) of the β chain (Fig. 7b) common with the GTP-bound complex but also additional interactions between T527 at α:L1, R593 at α:L2 of α subunit with M537, P538, R539 at the β:L2 region of the β subunit (Table 2). A possible reason for this protein-protein interaction rearrangement is the inability of β-hairpin loop 2 (β6L2β7) of α chain to engage with h2 of β chain opening the catalytic pocket which, in turn, results in the opening of the binding pocket.

### Per-residue decomposition of binding free energy

**Figure 8:**
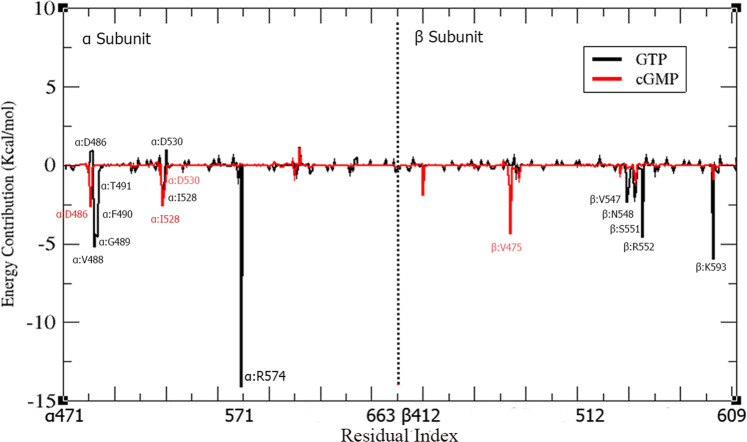
Per-residue MM-PBSA binding free energy contribution of the GTP-bound *h*sGC catalytic dimer (black) and cGMP-bound *h*sGC catalytic dimer (red). The residues showing the highest fluctuations are highlighted.

In order to further delineate the role of key residues that influence the overall binding free energy of the systems; per-residue energy decomposition analysis was performed for the identification of the interaction spectra of the GTP/cGMP-bound *h*sGC catalytic dimer. The last 20 ns snapshots of the 100 ns simulations were considered. The binding affinity of each residue in the ligand-receptor complex depends on the binding free energy (ΔG) contribution, which can be positive or negative. Negative ΔG values of a residue indicate higher binding affinity. Conversely, positive ΔG values are indicative of a repulsive effect that in turn doesn’t facilitate the catalytic activity of the receptor. In GTP-bound *h*sGC catalytic dimer, the most negative contribution to the binding free energy was observed for the residues spanning the following α and β subdomain regions of cyclase; α:h1 (V488, G489, F490, T491), α:β5 (R574), α:β2L1β3 (I528), and β:h4 (V547, N548, S551, R552), L3 (K593) (Fig. 8). These residues play a crucial role in the catalytic activity of the *h*sGC cyclase heterodimer. However, in the cGMP-bound *h*sGC catalytic dimer, a different pattern emerged, with α:β1 (D486), α:β2L1β3 (I528, D530), and β:β2L1β3 (V475) showing the most negative contribution to the binding free energy (Fig. 8). Table 3 depicts the average binding free energy of each residue in the GTP/cGMP-bound *h*sGC cyclase complexes. Interestingly, the D486 and D530 residues of the cyclase α subdomain showed negative ΔG values −2.60 Kcal/mol and −0.54 Kcal/mol, respectively, upon cGMP binding whereas, in case of GTP binding, the same residues showed repulsive effects. The key residues that contribute significantly to the binding of GTP in the GTP-bound *h*sGC catalytic dimer complex; α:R574 (−14.10 Kcal/mol), α:V488 (−5.18 Kcal/mol), α:G489 (−4.43 Kcal/mol), α:F490 (−4.50 Kcal/mol), β:F543 (−2.34Kcal/mol), β:V547 (−2.03 Kcal/mol), β:R552 (−4.60 Kcal/mol), and β:K593 (−6.00Kcal/mol), showed less negative or positive ΔG values in the cGMP-bound complex (Table 3). These per-residue energy decompositions findings are consistent with the binding mode analysis, which strengthens the argument that involvement of more residues in the case of the GTP-bound cyclase αβ complex contributes to the closed conformation of the binding pocket. The closed pocket structural arrangement is destabilized by the release of the pyrophosphate during the GTP to cGMP catalysis, leading to a decrease of the overall binding free energy and opening of the cyclase pocket.

**Table 3:** Per-residue energy decomposition (PRED) of GTP/cGMP-ligated *h*sGC cyclase systems.

Residues	Cyclase-GTP PRED ∆G(kcal/mol)	Cyclase-cGMP PRED ∆G (kcal/mol)
α:D486	−0.90	−2.60
α:V488	−5.18	0.01
α:G489	−4.43	−0.01
α:F490	−4.50	−0.01
α:T491	−0.37	0.01
α:I528	−2.50	−2.60
α:G529	−1.08	−1.60
α:D530	−1.00	−0.54
α:R574	−14.10	0.05
β:T474	0.05	−1.24
β:V475	−0.04	−4.40
β:F543	−2.33	−0.17
β:G544	−1.41	−0.10
β:N545	−0.22	−0.12
β:T546	−0.25	0.04
β:V547	−2.03	−0.60
β:N548	−1.71	−0.10
β:L549	−0.02	0.02
β:T550	−0.25	0.13
β:S551	−0.25	0.01
β:R552	−4.60	0.03
β:K593	−6.00	−1.00
β:K595	−0.40	−0.10
β:K596	−0.45	−0.10

### Principal Component Analysis (PCA)

**Figure 9:**
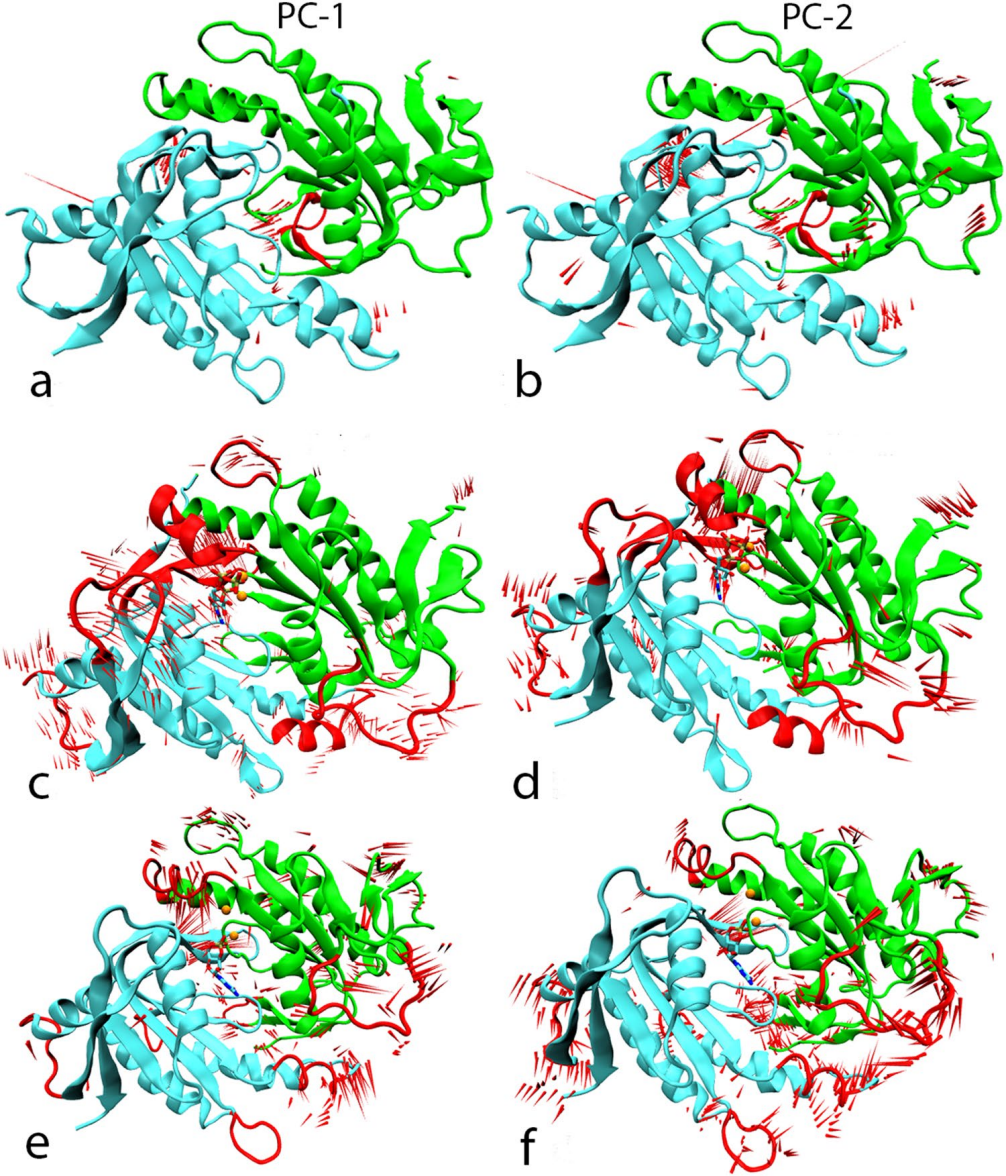
Porcupine plots of the first (PC-1) and second (PC-2) modes of motion of the apo (**a,b**) GTP-bound (**c,d**) and cGMP-bound (**e,f**) complexes of the hsGC catalytic dimer. Highly fluctuating regions are highlighted in red. The length of the red “needles” represents the degree of mobility.

We have applied principal component analysis to compare the overall conformational pattern of movements of the cyclase αβ heterodimer under the influence of GTP and cGMP, with the apo system. The trajectories of all systems (cyclase αβ-apo, cyclase αβ-GTP, cyclase αβ-cGMP) were processed to calculate eigenvectors that correspond to the dominant structural movements during a simulation. An essential dynamics approach was applied to the backbone atoms of all systems to understand their structural transitions. The most notable movements observed in the top two eigenvectors were the motion of α:h1, β-hairpin loop 2 (α:β6-β7), βh1, β-hairpin loop 2 (β:β6-β7), β:L3 regions and account for 85% of all the motions. Furthermore, the analysis assisted in understanding the conformational effect of cGMP and GTP on the cyclase dimer. The ProDy plugin^20^ integrated within VMD^21^ was employed to produce porcupine plots associated with the first two normal modes in each system (Fig. 9). The cyclase αβ-GTP complex showed substantial movement of the α:h1, β:β6-β7 and β:L3 regions towards the binding pocket of GTP probably due to the strong electrostatic interaction network generated upon ligand binding. The hydrogen bond analysis of the GTP-*h*sGC complex discussed above also suggests a cooperative effect at the dimerization interface further stabilizing the closed-state conformation upon GTP binding and facilitating the catalysis of GTP to cGMP. The cyclase αβ-cGMP system first and second principal components show a distinct pattern in which the α:h1, β:h1 and α:β6-β7 regions are more distant from the binding pocket, with particularly the β-hairpin loop of α:β6-β7 moving away from the dimer interface. The structural transition of these elements away from the binding pocket suggests a role in the post-catalysis structural dynamics. These findings are compatible with the stability analysis and earlier experimental studies^7,19,22^.

**Figure 10:**
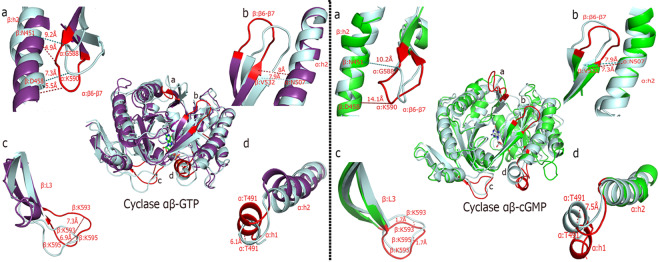
Fluctuations/deviations (Å) of important secondary structure elements a) β:h2, α:β6-β7 b) β:β6-β7, α:h2 c) β:L3 d) α:h1, α:h2 that govern the dynamics of the closed (GTP-bound, purple) and open (cGMP-bound, green) state of the *h*sGC catalytic heterodimer. Distances were calculated between the initial state (cyan) and maximum transition state of the aforementioned systems.

Furthermore, the structural transitions of the open (cGMP-bound) and closed state (GTP-bound) of the *h*sGC catalytic domain heterodimer are summarily illustrated in Fig. 10 by measuring the deviation of key residues and secondary structure elements from the reference/ initial state structure (docked GTP and cGMP-bound sGC cyclase) at the maximum transition points that were observed during PCA.

### Discussion and Conclusion

The structural assembly and activation of the soluble guanylate cyclase has been extensively studied by conventional biochemical studies^7,23,24^. Recently a cryo-EM-driven multi-domain quaternary structure of the human cyclase domain has been proposed^1^. Most studies discuss the molecular architecture of sGC in prokaryotes and eukaryotes^13,25^ with a few focusing on the molecular mechanism of the NO-based activation of sGC^26^ but little is known about the conformational changes within the cyclase domain upon GTP binding and catalysis^24,27^. The current study investigates the structural events that take place within the the *h*sGC cyclase domain during the binding and the cyclization event of GTP to cGMP.

The soluble guanylate cyclase is a core signaling molecule of the cGMP signaling pathway but biophysical and structural properties driven by GTP and cGMP binding to the cyclase domain remain only partially characterized^24,28^. We characterized the structural behavior of the cyclase domain upon GTP and cGMP binding. We applied MD simulations to investigate the transition between the open and closed state of the cyclase domain of *h*sGC. Since simulation of the actual transition from the open to the closed state conformation is particularly challenging, a targeted approach was adopted where three systems (apo, GTP- and cGMP-bound cyclase dimers) were studied concurrently and the fluctuations of the secondary structure elements that govern the opening and closing of the binding pocket were closely observed at the nanosecond scale. The structural dynamics of the apo, GTP- and cGMP-bound cyclase dimer complexes are compared with experimental data from the closely related adenylate cyclase catalytic dimer.

The catalytic dimer of adenylate cyclase is sharing a similar catalytic fold with the cyclase dimer of *h*sGC and most of the active site residues are highly conserved^27^. Among the catalytic residues, D440, D530 and E473 are interacting with GTP molecule^22^. A study by Agulló *et al*., 2016 reported that E473 located at the sGC cyclase β chain is essential for binding to the guanine moiety of the GTP molecule^29^. A role of glutamate residues in the selectivity and binding of GTP versus ATP molecules has also been proposed^30^.

In our simulated GTP-sGC catalytic dimer, E473 lies at a β-hairpin loop (β2L1β3) of the β subunit in the catalytic pocket. Additionally, D440 is present at β2 while D530 at β2L1β3 of the α chain of the sGC catalytic dimer. These secondary structure elements are part of the catalytic pocket assisting in the entrapment of GTP and cGMP. The dynamics of these secondary structure elements involved in the composition of active site have been shown to be substrate-specific^14^. In the current study, the GTP-bound cyclase domain showed a slightly inward movement of α: h1 which brings 489 G, 490 F, 491 T close to GTP. Strong hydrogen bond interactions with high occupancy were observed between 489 G, 490 F, 491 T and GTP. During the catalysis of GTP to cGMP, threonine residues have been shown to assist in guiding water to the γ-phosphate^31^. Theses α:h1 residues are not only binding the phosphate groups of GTP but also stabilizing the closed state of the GTP- sGC catalytic dimer.

Per-residue decomposition analysis complements the hydrogen bond analysis by highlighting the atomistic level energy contributions of active site residues in the ligand-bound states of the *h*sGC cyclase. In the closed state (GTP-bound) cyclase system, more residues interact with the ligand compared to the cGMP-bound state. Significant energy contributions of α:V488, α:G489, α:F490, α:R574, β:F543,β:V547,β:N548 and β:R552 were observed in the GTP-bound cyclase system, otherwise absent or opposite in the cGMP-bound system. Differences in the protein-protein interactions between intra-domain residues were also witnessed in the two systems under study. The binding free energy analysis results reinforce the molecular docking results proposed in the current study.

Similar structural transitions have also been proposed in other class III nucleotidyl cyclases due to ATP and GTP binding^19,32^. A catalytically active closed state of the GTP binding pocket is induced by the inward movement of h1 of α chain of the sGC catalytic dimer^14^. In all class III nucleotidyl cyclases members, GTP binding causes rotation of the α chain at its center translocating h1 close to h4 of the β chain (C2 domain of AC)^32^. In the h4 of the β chain, a conserved NSR motif is present. The serine residue is known to bind to the pentose sugar moiety of the GTP and cGMP^22^. Movement of α:h1 and β:h4 drives the protein to an active closed conformation which helps all the important catalytic residues to adopt orientations that support the cyclization event. The aforementioned conformational shift is communicated through the entire sGC catalytic dimer inducing β6L2β7 of the β subunit to close the active site pocket by interacting with h2 of the adjacent α subunit. This β6L2β7 movement also aids the stabilization of the GTP within the binding pocket through an interaction of L542 with the nitrogenous base of the GTP molecule. Movements of L1 (β3L1β4) and β6 (β6L2β7) in β chain are not only holding GTP in the center of the binding pocket through strong hydrogen bond interactions of E473 and L542 with GTP but also generating significant protein-protein interactions through the substrate-induced structural transitions further contributing to the closed state conformation of the sGC catalytic dimer. An inward shift of L3 of the β subunit towards the GTP binding pocket was also observed. L3 of the β subunit harbors K593 which binds with the phosphate group of GTP with significant hydrogen bond interactions and Lys residues in ATPases and GTPases have been shown to stabilize the pentavalent transition state of the phosphate during hydrolysis^33,34^.

In the cGMP-sGC catalytic dimer, due to the cleavage of γ-phosphate from GTP, the sGC catalytic dimer assumes an open conformation. Loss of a few interdomain interactions resulted in the detachment and outward movement of α:h1 from β:h4 and α:β6-β7 from the adjacent h2 of the β chain and subsequent opening of the active pocket. Outward movement of α:h1 and α:β6-β7 also resulted in the loss of electrostatic interactions of the nucleotide with E473, R574, G489, F490, T491, K593, N548 and R552.

Moreover, residues T491 and L542 located on α:h1 and β:β6-β7, respectively, establish robust electrostatic interactions with the GTP substrate, possibly facilitating the moderate movement of these regions inward while the absence of interactions in the cGMP-bound system results in the extrication of these regions and the opening of the binding cavity of the cyclase heterodimer. The understanding of the mechanistic basis of the substrate-specific opening and closing of the binding pocket in a more detailed manner was further aided by principal component analysis.

To conclude, we presented the substrate-specific and catalytically restrictive structural dynamics observed in the GTP- and cGMP-bound *h*sGC catalytic heterodimer. All observed conformational changes were validated with prior experimental findings and novel insights have been revealed from a comparative substrate-specific open and closed pocket conformation of the *h*sGC catalytic heterodimer. The findings of this study will be helpful in understanding the molecular mechanism underlying the GTP- and cGMP-induced structural changes and an aid future therapeutic intervention. Although all proposed observations are computationally simulated, they provide a basis for prioritization of future experimental approaches to establish a structural link between the closed and open state of the active pocket upon GTP and cGMP binding.

## Methodology

### Active site prediction of the *h*sGC cyclase domain

As the active heterodimer of the *h*sGC cyclase domain has not been structurally resolved yet, we applied a knowledge-based computational approach to identify the binding pocket for GTP and cGMP docking. For this purpose, the crystal structure of the heterodimeric *h*sGC cyclase domain in the apo-form solved at 2.08 Å was retrieved from the Protein Data Bank (PDB ID: 3UVJ)^14^ Allerston *et al*., 2013. demonstrated that the catalytic site of the *Rattus norvegicus s*AC catalytic dimer and the *hs*GC cyclase domain share the same structural fold and their catalytic residues are highly conserved^18^. Sequence alignment of the *Rattus norvegicus s*AC catalytic domain and the *h*sGC cyclase domain was performed with the CLC Genomics workbench^35^ to assess the highly similar/identical sequence regions. The catalytic dimer of the ATP-bound *Rattus norvegicus s*AC was superposed to the *h*sGC cyclase dimer (PDB ID: 3UVJ) in the Schrödinger software *(Schrödinger Release 2018-3)* to locate the binding pocket of the *hs*GC cyclase domain^36^. The active site residues of the dimeric interface were precisely superimposed. For docking purposes, the catalytic site of the *h*sGC cyclase domain was used as a receptor site for molecular docking.

### Protein preparation

Before docking, the retrieved crystal structure of the heterodimeric *h*sGC cyclase domain in the apo-form from the Protein Data Bank (PDB ID: 3UVJ) was prepared in Schrödinger’s (Schrödinger Release 2018-3) multi-step Protein Preparation Wizard^36^. Protein preparation involved addition of all missing hydrogen atoms and removal of water molecules and any heteroatoms. In addition to this, bond orders were assigned and protonation states were then adjusted. To minimize steric hindrance between the atoms, restrained energy minimization was performed by applying the Optimized Potential for Liquid Simulations 2005 (OPLS 2005) force field^37^.

### Molecular docking

The Glide XP (extra precision) module of Maestro^38,39^ was selected for the docking of GTP and cGMP to the receptor protein. To validate the docking algorithm, ATP was re-docked to the binding cavity of the *R. novergicus* sAC catalytic domain. Based on the centroid of the already bound ATP within the active site of *R. novergicus* sAC catalytic domain, a grid box of specific dimensions was generated.

The GTP and cGMP molecules were prepared using the Schrödinger 3D builder of (Schrödinger Release 2018-3) and minimized using the OPLS3e force field^40^. To dock GTP and cGMP within the dimeric active site of *h*sGC, the grid box dimensions (grid size ≤ 16 Å) of the *R. norvegicus* catalytic domain used for the ATP docking were applied. Two-dimensional (2D) ligand interaction diagrams were generated with Intermezzo (Ochoa-Montaño B, Blundell TL – unpublished).

### Substrate parametrization

Accurate force fields are vital for generating the dynamic and conformational response of condensed-phase systems. The force field parameters for GTP were taken from Amber force field database^41^ while the parameterization of cGMP was done by applying quantum mechanics calculations through Gaussian using the Hartree Fock method and the 6–31 G* basis set^42^. The restrained electrostatic potential (RESP) charges of non-standard residues were calculated with the antechamber17 module^43^.

### MD system preparation

The cyclase-α_1_β_1_ (apo), cyclase-α_1_β_1_ GTP/cGMP-bound systems were subjected to all-atom MD simulations in explicit solvent employing the GPU version of the PMEMD engine provided with Amber14^44^. The receptor protein was parameterized with the ff14SB force field^45^. The tleap program was applied to add missing hydrogen atoms and counter ions for neutralizing the systems. All three complexes were immersed in an orthogonal box with a TIP3P water model and periodic boundary conditions^46^. The long-range electrostatic interactions were calculated with the Particle Mesh Ewald (PME) method with a vdW cut-off of 10 Å^47^. After the completion of parametrization, the geometry of all prepared systems was minimized (20000 steps) to remove steric hindrances. The complexes were heated from 0 to 300 K at constant volume with a Langevin thermostat (200 ps). Subsequently, productive MD simulations at constant pressure (NPT) were conducted for each complex for, at least, 100 ns. During the entire MD run, the covalent bonds bearing hydrogens were restrained applying the SHAKE algorithm^48^. Trajectory snapshots were acquired every 2 fs.

### Stability analysis

Trajectories taken from all MD simulations were interpreted with the help CPPTRAJ v17.00 module^49^. The Root Mean Square Deviation (RMSD) was calculated for all backbone atoms with the starting structure as a reference frame. The Root Mean Square Fluctuation (RMSF) of the backbone atoms over the entire simulation was also calculated. The overall compactness of the structures was assessed with the radius of gyration (Rog) calculation. Stability analysis plots were generated using Xmgrace^50^.

### Hydrogen bond occurrence

Hydrogen bonding plays a critical role in folding and the wreath-like quaternary packing of the *h*sGC catalytic subdomains. Therefore, changes in the hydrogen bond occupancy pattern possibly contribute to different aggregation propensity and folding of the cyclase αβ heterodimer. Hydrogen bonds of the different conformational arrangements of the cyclase αβ heterodimer in the presence of GTP and cGMP substrates were analyzed by considering the distance between the heavy atoms and the angle between the acceptor and donor atoms. The occurrence of hydrogen bonds was visualized with the Ligplot^+^ tool^51^ which depicts the most persistent hydrogen bond interactions over the course of the 50 ns MD simulations.

### MM-PB/GBSA calculations

The Molecular Mechanics with Poisson Boltzmann/Generalized Born and Surface Area solvation MM-PB/GBSA protocol was used to estimate the nucleotidyl (GTP/cGMP) binding free energy (ΔGbind) against the cyclase-αβ heterodimer by considering the interacting molecules solvation energy along with molecular mechanics (MM) energies. The amber14 MMPBSA.py module^52^ was implemented for the estimation of MM-PB/GBSA. The program calculates multiple energy terms such as electrostatic energy, van der Waals interaction energy, polar and non-polar solvation free energy. In addition, the binding free energy of each residue was also calculated in the aforesaid energy terms. This approach assists in identifying critical residues and helps in reproducing their relative binding affinities to a given set of ligands. Overall, binding free energy (ΔGbind) of a ligand-receptor complex can be estimated by assessing the energy of three reactants with these equations^53^ Eq. 1

1 $$\Delta {\rm{Gbind}}={\rm{avg}}\,{\rm{G}}\,{\rm{complex}}-{\rm{avg}}\,{\rm{G}}\,{\rm{receptor}}-{\rm{avg}}\,{\rm{G}}\,{\rm{ligand}}$$

where avg indicates an average over an ensemble of trajectory snapshots, and G the free energy contributions of the complex, the receptor and the ligand. Therefore, the binding energy of the reactant is calculated with Eq. 2.

2 $${\rm{G}}={\rm{MM}}+{\rm{G}}\,{\rm{solvent}}-{\rm{TS}}\,{\rm{solute}}$$

where MM term corresponds to the molecular mechanics contribution in vacuum, comprising of the aggregate of internal, van der Waals and electrostatic energies; G solvent corresponds to the solvation free energy delineated in terms of total polar and non-polar solvation free energy; whereas TS solute explores the temperature and solute entropy respectively^54^. In order to investigate the per-residue binding energy of GTP/cGMP bound cyclase-αβ complexes, snapshots of the last 20 ns were extracted from the trajectories of 100 ns, and the average binding free energies over the collection of conformers was calculated. The GB and PB methods for the estimation of polar solvation energies were applied with default settings. The non-polar solvation free energy was determined by measuring the solvent-accessible surface area^55^.

### Essential dynamics

Principal component analysis (PCA) was carried out to identify and distinguish the significant structural transition modes in the cyclase-αβ apo-form, GTP- and cGMP-bound complexes by utilizing MD trajectory data. This method works by constructing a covariance matrix of the backbone structural transitions ignoring the overall rotational and translational movements^56^. The diagonalization of the covariance matrix results in the generation of structurally uncorrelated variables corresponding to certain eigenvectors (eigenvalues). These eigenvalues are arranged in decreasing order where the first principal component (PC-1) is associated with the largest structural transitions of the proteins. PCs of all three systems were obtained with the CPPTRAJ module of Amber14. In all three systems, the first three PCs contributed the majority of structural transitions over the entire simulation. The Visual Molecular Dynamics (VMD) tool was employed to comprehensively investigate the uncorrelated system transitions and the Normal Mode Wizard (NMWiz) was used to create porcupine plots^20^. Graphical representations were constructed with PyMol and VMD^57^.

## Supplementary information

Supplementary Information.
