# Supplementary material for: Probing the Structural Dynamics of the Catalytic Domain of Human Soluble Guanylate Cyclase
Source: Sci Rep. 2020 Jun 11;10:9488. doi: 10.1038/s41598-020-66310-4 (PMC7289801; doi:10.1038/s41598-020-66310-4)
Supplement: Supplementary file 1 — Supplementary Information. [file 41598_2020_66310_MOESM1_ESM.docx]

**Supplementary Material**

**For**

**Probing the Structural Dynamics of the Catalytic Domain of Human Soluble Guanylate Cyclase**

Rana Rehan Khalid^a,b,c^, Arooma Maryam^a,d^, Osman Ugur Sezerman ^c^, Efstratios Mylonas ^e^, Abdul Rauf Siddiqi^a^ ^*^,Michael Kokkinidis ^e^*

^a^ Department of Biosciences, COMSATS University, Islamabad 45550, Pakistan; [ray.binm@gmail.com](mailto:ray.binm@gmail.com)(R.R.K); [aroomabinm@yahoo.com](mailto:aroomabinm@yahoo.com) (A.M)

^b^ Department of Biology, University of Crete, 70013 Heraklion, Greece

^c^ Department of Biostatistics and Medical Informatics, Acibadem Universitesi, Istanbul 34752, Turkey

^d^Department of Pharmaceutical Chemistry, Biruni Universitesi, Istanbul 34010, Turkey

^e^Institute of Molecular Biology and Biotechnology, Foundation for Research and Technology-Hellas (IMBB-FORTH), 70013 Heraklion, Greece; [stratos_mylonas@imbb.forth.gr](mailto:stratos_mylonas@imbb.forth.gr) (E.M)

*Correspondence: kokkinid@imbb.forth.gr (M.K); abdulraufsher@gmail.com (A.R.S.); Tel.: ++30-6944-362871 (M.K); +92-300-5253299 (A.R.S.)

**
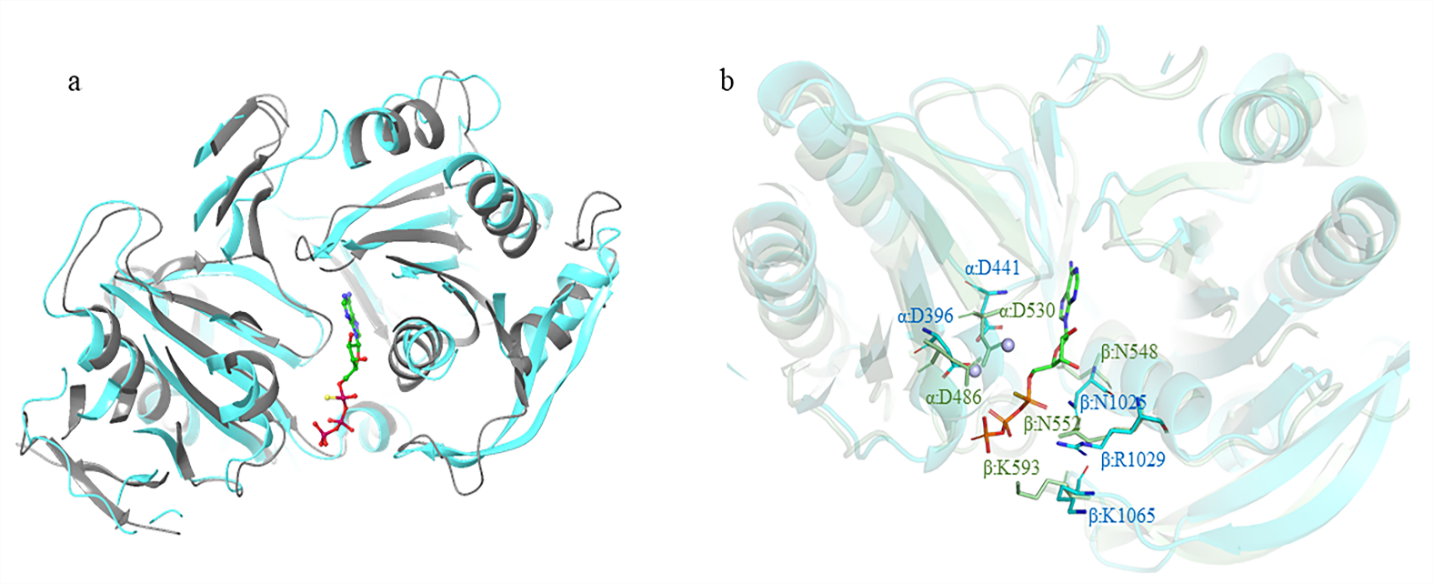
**

**Supplementary Figure 1.** a) Structural superposition of the human soluble guanylate cyclase dimer (hsGC) with the closely related ATP-bound R.norvegicus adenylate cyclase dimer (sAC). R.norvegicus sAC is shown in cyan while hsGC dimer is shown in grey. b) Highlighting the conserved residues of the binding pocket of the ATP-bound R.norvegicus AC (cyan) and hsGC dimer (green).
